# Estrogen suppresses SOX9 and activates markers of female development in a human testis-derived cell line

**DOI:** 10.1186/s12860-020-00307-9

**Published:** 2020-09-15

**Authors:** Melanie K. Stewart, Deidre M. Mattiske, Andrew J. Pask

**Affiliations:** School of BioSciences, The University of Melbourne, Parkville, VIC 3010 Australia

**Keywords:** Estrogen, Sertoli cells, Testis, Sex determination

## Abstract

**Background:**

The increasing incidence of reproductive disorders in humans has been attributed to in utero exposure to estrogenic endocrine disruptors. In particular, exposure of the developing testis to exogenous estrogen can negatively impact male reproductive health. To determine how estrogens impact human gonad function, we treated the human testis-derived cell line NT2/D1 with estrogen and examined its impact on SOX9 and the expression of key markers of granulosa (ovarian) and Sertoli (testicular) cell development.

**Results:**

Estrogen successfully activated its cognate receptor (estrogen receptor alpha; *ESR1*) in NT2/D1 cells. We observed a significant increase in cytoplasmic SOX9 following estrogen treatment. After 48 h of estrogen exposure, mRNA levels of the key Sertoli cell genes *SOX9, SRY, AMH, FGF9* and *PTGDS* were significantly reduced. This was followed by a significant increase in mRNA levels for the key granulosa cell genes *FOXL2* and *WNT4* after 96 h of estrogen exposure.

**Conclusions:**

These results are consistent with estrogen's effects on marsupial gonads and show that estrogen has a highly conserved impact on gonadal cell fate decisions that has existed in mammals for over 160 million years. This effect of estrogen presents as a potential mechanism contributing to the significant decrease in male fertility and reproductive health reported over recent decades. Given our widespread exposure to estrogenic endocrine disruptors, their effects on SOX9 and Sertoli cell determination could have considerable impact on the adult testis.

## Background

Differences of sexual development (DSDs) are currently some of the most common birth defects in humans and often affect gonadal development and fertility. DSDs occur in 1:200–1:300 live births [1] and are increasing in incidence [2, 3]. In Western countries in particular, DSDs continue to rise while sperm counts have decreased by more than 50% in the last 40 years [4]. This sharp decline in male reproductive health is of major concern and there is an urgent need to identify and understand potential contributors to this issue.

Although infertility and DSDs can have genetic causes, the recent and rapid decline in overall reproductive health and fertility is now unequivocally linked to our exposure to endocrine disrupting chemicals (EDCs) [5–10]. Estrogenic EDCs are some of the most pervasive in our environment and include compounds such as bisphenol A, 17α-ethynylestradiol, phthalates, and genistein. These EDCs are capable of interacting with estrogen receptors to trigger ectopic activation of estrogen responsive signaling pathways [11]. Elevated estrogen levels are known to negatively impact the adult testis, leading to infertility [12]. Furthermore, estrogen signaling is imperative during fetal development, where the correct balance of androgens and estrogens is required for sexual differentiation of both the male and female reproductive tracts [13–15].

The role of estrogen in directing vertebrate gonad development has been well studied. In non-mammalian vertebrates, estrogen plays a fundamental role in actively directing the indifferent gonad towards an ovarian fate [16]. Estrogen is also essential for gonad differentiation in many mammalian vertebrates, such as in goats [17], sheep [18, 19], and cows [20], where production of the enzyme aromatase promotes the synthesis of estrogen from testosterone in the fetal ovary. In marsupials, exposure of embryonic XY gonads to exogenous estrogen before sex determination results in complete ovarian formation in the opossum [21], and tammar wallaby [22], demonstrating the ability of the hormone to promote ovarian fate despite genetic predisposition to form a testis.

Estrogen receptors (ERs) are expressed in the indifferent somatic cells of the fetal gonad in all mammals and are targets of estrogen during critical periods of development [23–25]. The human XX indifferent gonad produces estrogen before ovarian differentiation [26], suggesting it may have a function in driving ovarian development.

In contrast, estrogen is not thought to play a direct role in mouse fetal ovarian formation as deletion of ERs or aromatase (encoded by *Cyp19*) does not affect early ovarian differentiation [27, 28]. Shortly after birth, somatic cells in ER- or *Cyp19*-deficient ovaries upregulate *Sox9* and take on Sertoli cell morphology, demonstrating estrogen signaling is required for granulosa cell and ovarian maintenance in mice. When exogenous estrogen is administered, these ‘Sertoli-like’ cells downregulate *Sox9* and granulosa cell fate is rescued [27, 28]. Thus, in rodents, estrogen plays a central role in maintaining granulosa cell fate and repressing key testis factors such as *Sox9*.

*Sox9* is both necessary and sufficient to drive testicular development [29]. Prior to embryonic day (E) 11.5 in mice, SOX9 is present in the cytoplasm of somatic cells in the gonads of both sexes [30]. At E11.5, when *Sry* reaches a peak in expression in XY embryos, SOX9 is rapidly translocated to the nucleus where it triggers a cascade of transcriptional events leading to testis determination. Specifically, it upregulates expression of itself and the key testis genes, *Amh, Fgf9,* and *Ptgds,* among others [31].

In females, the absence of *Sry* leads to the disappearance of the cytoplasmic pool of SOX9 and transcription of *Sox9* ceases. This allows for the stabilisation of β-catenin, which is essential for ovarian formation [32] and expression of the ovarian genes *Foxl2* and *Wnt4. Wnt4* is present in both XX and XY gonads at E11.0 but is downregulated in males at E11.5 [33]. *Foxl2* is necessary for maintenance of granulosa cell fate and its ablation in mouse ovaries leads to transdifferentiation of granulosa cells to a Sertoli cell phenotype with upregulation of *Sox9* [34]. FOXL2, in conjunction with activated estrogen receptors, is proposed to maintain granulosa cell fate by suppressing *Sox9* transcription. This demonstrates the plasticity of these cells and the ‘push-and-pull’ between testis and ovarian markers, which can be mediated in part by estrogen. Furthermore, these studies illustrate that the translocation of SOX9 into the nucleus is a critical step for the formation of a testis and repression of ovarian development.

In marsupials, estrogen can impact two of the fundamental transcription factors in regulating gonadal cell fate decisions: SOX9 and FOXL2 [34, 35]. Exogenous estrogen suppresses SOX9 by trapping it in the cytoplasm of somatic cells. This prevents upregulation of male specific pathways and induces expression of *FOXL2*, indicative of granulosa cell fate [35]. In humans, nuclear SOX9 is similarly required for male development and mutations that affect SOX9 nuclear import result in DSDs [36–38]. However, it is unknown if estrogen can impact the subcellular localisation of SOX9 in humans. In the present study, we exposed the human testis-derived cell line NT2/D1 to estrogen and examined its impact on the subcellular localisation of SOX9.

## Results

### Estrogen activates estrogen receptor α in NT2/D1 cells

**Fig. 1:**
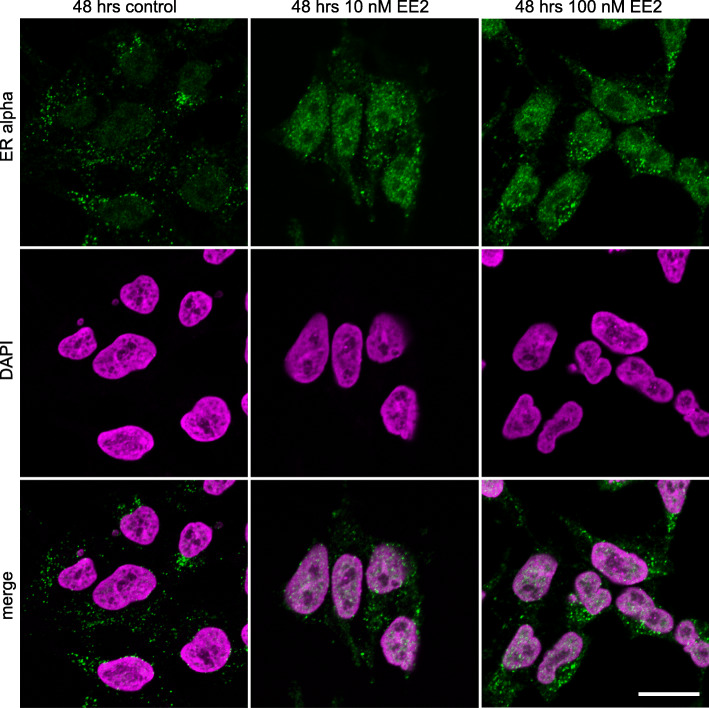
ERα immunofluorescence staining in NT2/D1 cells after 48 h of culture. ERα (green) was primarily cytoplasmic in control cells. EE2 treatment at both 10 nM and 100 nM led to a significant increase in nuclear ERα. Top panel shows ERα, middle panel shows DAPI, bottom panel shows a merge. Nuclei stained with DAPI; magenta. Scale bar 5 μm

Estrogen receptor α (ERα) was translocated to the nucleus at both concentrations of EE2 after 48 h. In comparison, in control cultures ERα remained largely cytoplasmic (Fig. 1). Nuclear localisation indicates the activation of ERα in the presence of both 10 and 100 nM of EE2 in our culture system and confirms the ability of the NT2/D1 cells to respond to estrogen exposures.

### Activated ERα increases cytoplasmic SOX9 levels

**Fig. 2:**
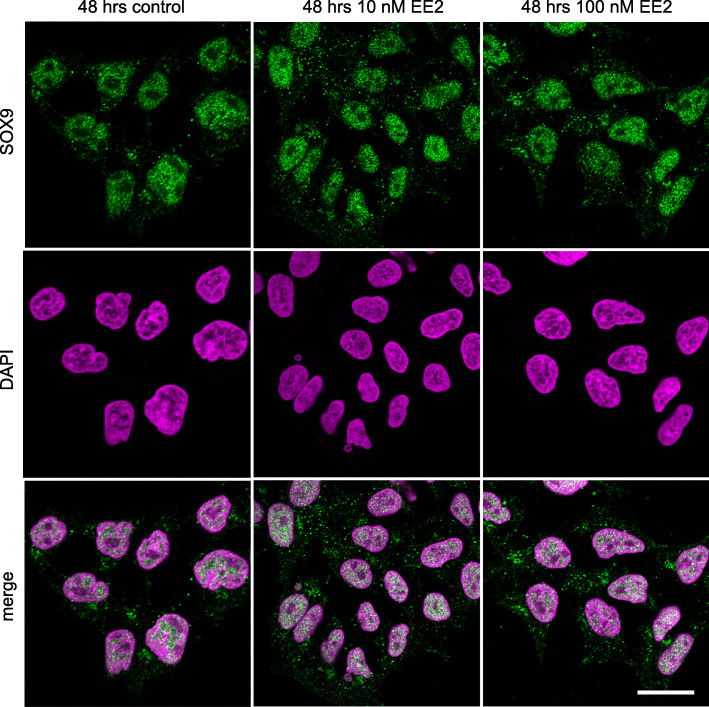
SOX9 immunofluorescence staining in NT2/D1 cells after 48 h of culture. SOX9 (green) was primarily nuclear (nuclei marked by DAPI; magenta) in the control cells. EE2 treatment at both 10 nM and 100 nM led to a greater amount of SOX9 in the cytoplasm. Top panel shows SOX9, middle panel shows DAPI, bottom panel shows a merge. Scale bar 5 μm

**Fig. 3:**
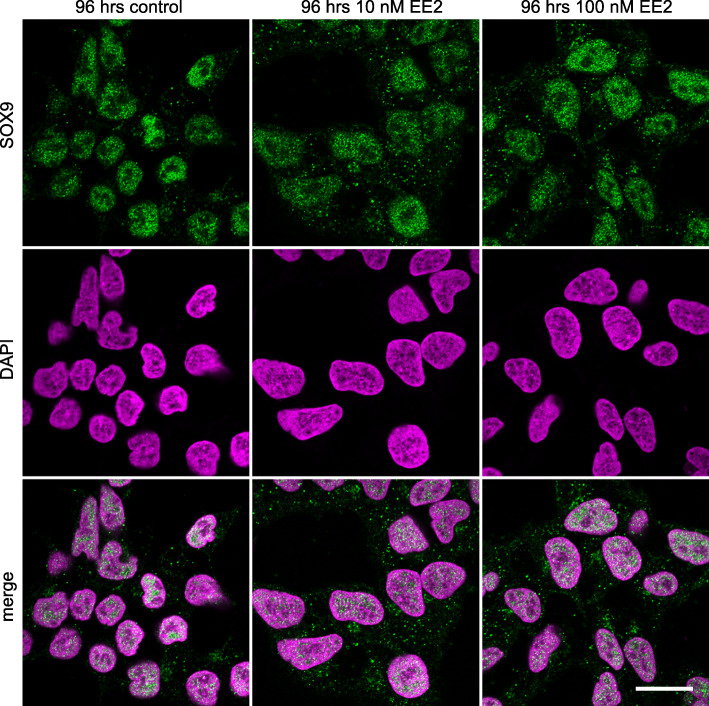
SOX9 immunofluorescence staining in NT2/D1 cells after 96 h of culture. SOX9 (green) was primarily nuclear (nuclei marked by DAPI; magenta) in the control cells. EE2 treatment at both 10 nM and 100 nM led to a greater amount of SOX9 in the cytoplasm. Top panel shows SOX9, middle panel shows DAPI, bottom panel shows a merge. Scale bar 5 μm

**Fig. 4:**
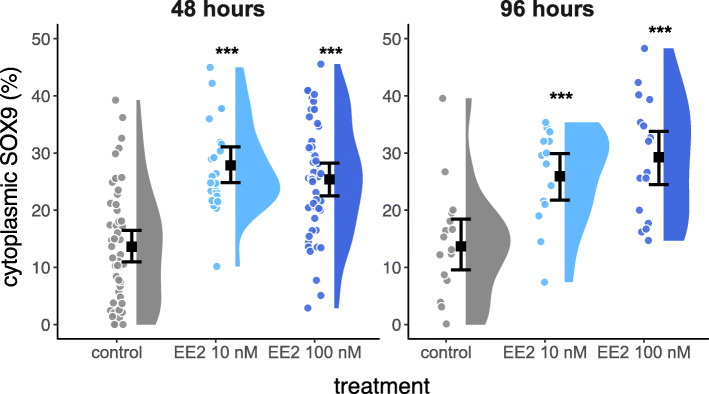
Raincloud plot of the percentage of cytoplasmic SOX9 in NT2/D1 cells. Each filled circle indicates a data point. The error bars delineate the 95% confidence interval of the mean (black square). The density distribution is indicated by the filled area. The percentage of cytoplasmic SOX9 is significantly increased for all treatments (* indicates *P* < 0.05, ** indicates *P* < 0.01, *** indicates *P* < 0.001 compared to the control)

SOX9 was primarily nuclear in control cultures of NT2/D1 cells, with a small proportion of cytoplasmic SOX9. SOX9 was still present in the nucleus in all estrogen treated cells, however, there was an increase in the amount of SOX9 in the cytoplasm after 48 (Fig. 2) and 96 h (Fig. 3) of culture. This was quantified by colocalisation analyses and shown in Fig. 4 (48 h: 10 nM EE2, *P* < 0.0001; 100 nM EE2, *P* < 0.0001; 96 h:10 nM EE2, *P* = 0.001; 100 nM EE2, *P* < 0.001). There were no significant differences in the amount of cytoplasmic SOX9 between estrogen concentrations (48 h: *P* = 0.37; 96 h: *P* = 0.41) or duration of treatment (10 nM, *P* = 0.26; 100 nM: *P* = 0.75).

### Estrogen suppresses testis markers after 48 h

**Fig. 5:**
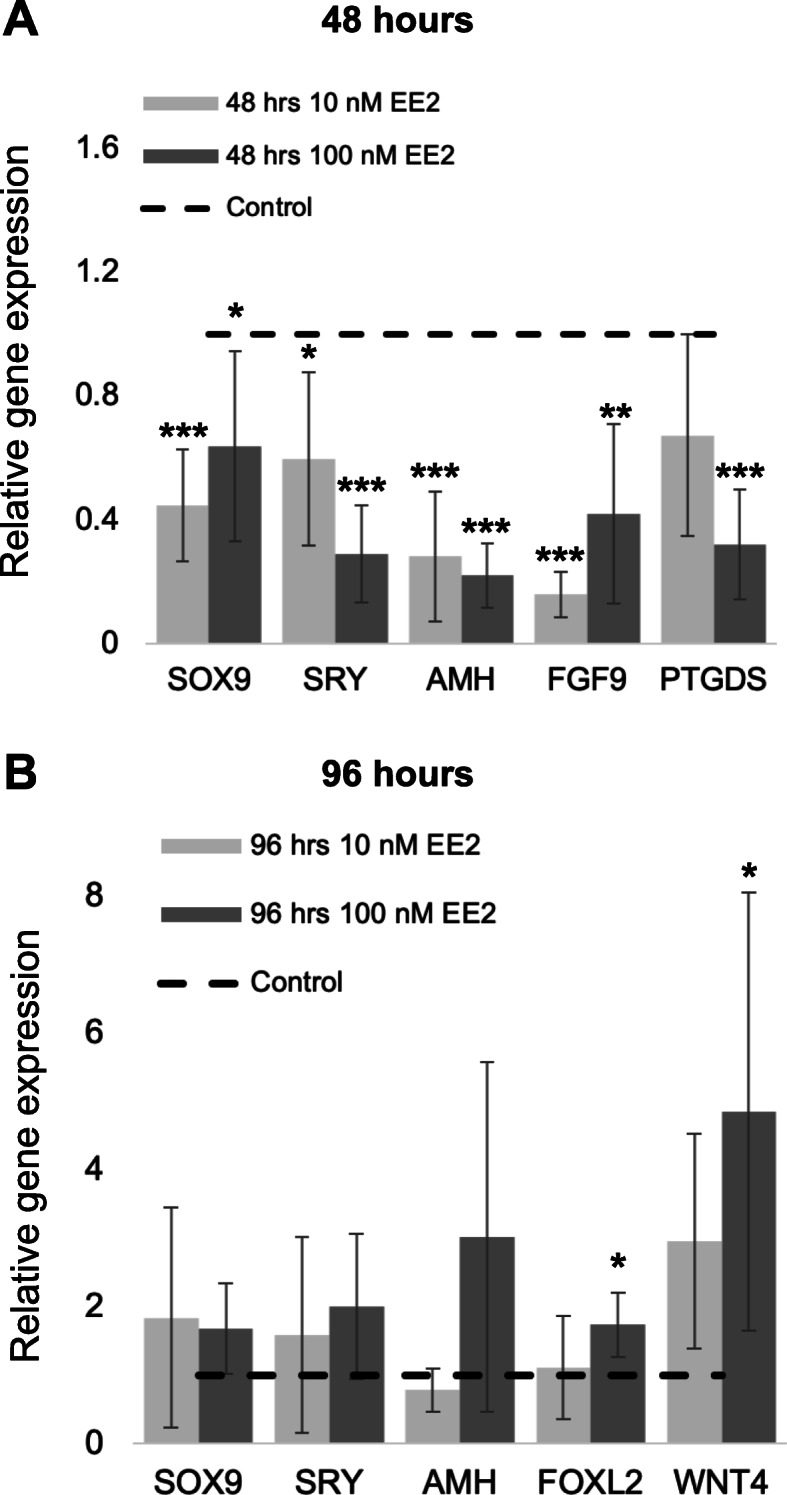
Gene expression of key gonad markers in NT2/D1 cells. Quantitative mRNA levels of *SOX9*, *SRY*, *AMH*, *FGF9* and *PTGDS* after 48 h (A; *n* = 10) and *SOX9*, *SRY*, *AMH*, *FOXL2* and *WNT4* after 96 h (B; *n* = 5) with either 10 nM or 100 nM of EE2. Expression levels in control cells were designated as 1 and illustrated by the dotted line. Error bars show the 95% confidence interval of the mean. Expression of each gene in the corresponding estrogen treated culture was expressed relative to control levels. All testis markers had significantly reduced mRNA levels following 48 h of EE2 treatment (**a**). Testis markers did not show reduced mRNA levels after 96 h, however the ovarian markers *FOXL2* and *WNT4* had significant increases in expression (**b**). (* indicates *P* < 0.05, ** indicates *P* < 0.01, *** indicates *P* < 0.001 compared to the control)

Estrogen treatment of NT2/D1 cells for 48 h (*n* = 10 cultures) reduced the mRNA levels of key testicular genes (Fig. 5a). The lower concentration of 10 nM of EE2 caused a significant reduction in mRNA levels of *SOX9* (*P* < 0.001)*, SRY* (*P* = 0.04)*, AMH* (*P* < 0.001) and *FGF9* (*P* < 0.001). *PTGDS* expression was also suppressed, but this was not significant (*P* = 0.1). One hundred nM of EE2 significantly reduced mRNA levels of *SOX9* (*P* = 0.016)*, SRY* (*P* < 0.001)*, AMH* (*P* < 0.001)*, FGF9* (*P* = 0.002)*,* and *PTGDS* (*P* < 0.001).

### Estrogen promotes female markers after 96 h

Prolonged estrogen treatment for 96 h (*n* = 5 cultures) led to activation of key ovarian genes (Fig. 5b). Cells treated with 10 nm of EE2 had an increase in mRNA levels of *WNT4*, however this was not significant (*P* = 0.06). *FOXL2* expression remained unchanged at this concentration. However, treatment with 100 nm of EE2 significantly increased the mRNA levels of *FOXL2* (*P* = 0.01) and *WNT4* (*P* = 0.04). Despite being significantly suppressed at 48 h, the male markers *SRY*, *SOX9* and *AMH* showed no significant suppression following 96 h of estrogen exposure (Fig. 5b).

## Discussion

Estrogen plays a critical role in sex determination and early sexual differentiation in all non-mammalian vertebrates [39]. However, the impact of estrogen on these processes in mammals has remained less clear. In marsupials, exposure of the developing gonads to exogenous estrogen blocks SOX9 nuclear import and affects its function at both the gene and protein level [35]. The nuclear translocation of the transcription factor SOX9 is essential for Sertoli cell specification and the formation of a testis [40]. Indeed, mutations affecting its nuclear import significantly impact testis development and cause differences in sexual development (DSDs) in humans [36, 38]. This study examined if exogenous estrogen could have an effect on SOX9 localisation in human cells potentially impacting its role to activate and maintain Sertoli cell fate.

We have used the human testis-derived cell line NT2/D1 to investigate the impacts of exogenous estrogen on SOX9 localisation and the downstream changes to genes regulating testicular and ovarian fate. NT2/D1 cells display cellular characteristics of embryonic Sertoli cells and express the repertoire of genes observed in testis determination [41, 42]. They have been used in numerous previous studies to elucidate the molecular mechanisms involved in key gonad pathways, including the factors involved in SOX9 nuclear import [43, 44], SOX9 regulation of *AMH* [45], regulation of SOX9 by PGD2 [46], interactions between SRY and β-catenin [47], mediators of SRY subcellular localisation [48], and the role of MAP3K1 in regulating gonadal pathways [49].

We observed a change in ERα distribution within NT2/D1 cells following estrogen exposures, with considerable accumulation in the nucleus. The nuclear localisation of ERα confirms its activation by estrogen [50]. This coincided with a significant accumulation of SOX9 in the cytoplasm of estrogen treated NT2/D1 cells, consistent with previous findings in marsupials [35]. Despite the accumulation of SOX9 in the cytoplasm, estrogen exposed cells still had some nuclear SOX9, but this was not sufficient to maintain the male development program after 48 h of estrogen treatment. Similarly, humans who are haploinsufficient for *SOX9* display male to female sex reversal despite having normal nuclear translocation of the protein [51], indicating that a threshold of SOX9 is required to activate the male development pathway.

Estrogen exposures caused a significant decrease in expression of the SOX9 target genes *AMH, PTGDS* and *FGF9* after 48 h. Nuclear SOX9 drives *AMH* upregulation in the developing testis of humans [45] to trigger the regression of the Müllerian ducts, facilitating patterning of the male urogenital tract [52]. Prostaglandin D synthase (*PTGDS*) is another downstream target of SOX9 and produces prostaglandin D2 (PGD2) [53], which is continually required to provide sustained expression of *SOX9* in the testis [54] and can induce nuclear import of SOX9 [44]. We also examined the impact of estrogen on *FGF9*, another key factor in maintaining *SOX9* expression and Sertoli cell differentiation in the developing testis [55]. Together, our results indicate that estrogen is able to impact the ability of SOX9 to activate the male developmental pathway in a human cell line.

Estrogen exposure also caused the induction of female marker genes, *WNT4* and *FOXL2* after 96 h of exposure. This is an identical response to that seen in the developing marsupial XY gonad [35], indicating a highly conserved role for estrogen in the initiation of the female developmental genes in mammals. The significant increase in *WNT4* mRNA levels was consistent with a concomitant significant decrease in *FGF9* expression. WNT4 and FGF9 are antagonistic signals in early gonad development, promoting ovarian and testicular fate respectively in the developing somatic cells [56]. This antagonistic relationship is conserved across humans, mice and marsupials [57]. *WNT4* is also known to be upregulated by estrogen in the human uterus and rat brain [58, 59]. Similarly, *Foxl2* is upregulated by estrogen in the adult mouse ovary [34] and is critical for ovarian development and the maintenance of granulosa cell identity [60].

Despite the clear induction of granulosa cell markers in NT2/D1 cells after 96 h of estrogen exposure, we also saw the reversion of *SRY* and *SOX9* expression to control levels. Since these developmental programs are mutually exclusive in gonadal somatic cells [34, 61, 62], these data suggest a mixed response of cells in our culture system, with some cells heading towards a granulosa-like fate while others are reverting back to a Sertoli-like state. This is also supported by the variation seen in our colocalisation results at 96 h, which shows that despite SOX9 mRNA levels returning to control levels after 96 h, there is still significantly more protein in the cytoplasm of the estrogen exposed cells. This reflects what is observed in a biological context, where the presence of paracrine factors (FGF9 and PGD2) promotes recruitment of neighboring cells to Sertoli cell fate and away from a granulosa cell fate [63, 64]. These results are also in line with what is observed in humans, where exposure to exogenous oestrogen negatively impacts male gonad development and fertility but does not cause complete sex reversal [12].

Cells treated for 96 h with the high concentration of estrogen also showed an increase in *AMH* expression, although this was not significant. While AMH is a direct downstream target of SOX9 in Sertoli cells, it is also expressed in granulosa cells, where it has a role in supporting folliculogenesis [65, 66]. *AMH* is upregulated in response to estrogen in mature granulosa cells [67], unlike in Sertoli cells where estrogen suppresses its transcription [this study, 35]. It is possible that as NT2/D1 cells transition towards a female fate after prolonged estrogen exposure, that AMH is positively responding to estrogen as it would in a normal female somatic cell. This interesting result could suggest estrogen is not only impacting its direct transcriptional targets but is also altering the regulatory landscape within cells.

## Conclusions

**Fig. 6:**
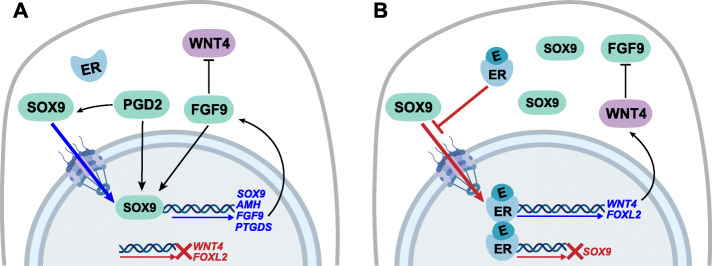
Model for estrogen regulation of SOX9 in Sertoli cells. **a** In a normal Sertoli cell SOX9 increases expression of itself and its downstream targets *AMH, FGF9, PTGDS* by translocating from the cytoplasm to the nucleus. PGD2 facilitates the nuclear entry of SOX9, while FGF9 inhibits WNT4 and there is no expression of *FOXL2*. **b** Exogenous estrogen (E) blocks SOX9 nuclear entry, increasing the amount of SOX9 in the cytoplasm and preventing activation of downstream SOX9 targets. Activated estrogen receptors (ERs) repress SOX9 transcription and promote expression of *WNT4* and *FOXL2*. WNT4 subsequently inhibits FGF9

Together, our data demonstrate that estrogen can alter the bioavailability of SOX9 within NT2/D1 cells to cause a suppression of the testis developmental program and promote transcription of key ovarian genes in a human testis-derived cell line (Fig. 6). These results are consistent with the known impacts of estrogen on the developing gonads in marsupials [21, 22, 35], indicating that estrogen may have a conserved role in mediating somatic cell fate across mammals. Furthermore, they implicate a role for estrogen in reinforcing the balance between female and male factors that maintain somatic cell fate in the gonad. This has broad implications for interpreting the potential effects of estrogenic endocrine disruptors on human development. We propose that the cytoplasmic retention of SOX9 is a potential mechanism for how estrogenic endocrine disruptors may be impacting Sertoli cells and testis development. Even a brief, reversible disruption to Sertoli cell function in the developing male fetus could have impacts on the patterning of the adult testis. The effects we have observed are significant but subtle in magnitude. This is consistent with the impacts of EDCs on humans where estrogen exposures cause morphological disruptions to the testis but not complete sex reversal in adult men [12]. Given the widespread exposures to estrogenic EDCs and the rapid increase in DSDs in humans, this effect of estrogen furthers our understanding about how such chemicals could be contributing to the overall decrease in male fertility and reproductive health.

## Methods

### Cell culture and estrogen treatment

The human testis-derived embryonal carcinoma NTERA-2 clone D1 [NT2/D1] (cells were purchased from ATCC.org; ATCC CRL-1973) cell line shares many properties with Sertoli cells. They express *SOX9*, show activation of SOX9 target genes, suppression of female markers, and endogenously express estrogen receptors [41, 42]. The cells were maintained at 37 °C, 5% CO_2_ in phenol red free Dulbeccos Modified Eagles Medium (to avoid the estrogenic effect of phenol red in culture [68]) supplemented with 10% fetal bovine serum (Gibco) and antibiotic-antimycotic (Gibco). Cells were either cultured on Nunc Lab-Tek Chamber slides (immunofluorescence experiments) or 6-well plates (qPCR experiments). Cells were treated with 17α-ethynylestradiol (EE2; Sigma Aldrich) dissolved in ethanol, for 48 h or 96 h at either 10 nM or 100 nM. EE2 was chosen due to its longer half-life in culture and its biological relevance given its widespread use and pervasiveness in our environment [69, 70]. The final concentration of ethanol added to culture was < 0.05%. An identical amount of ethanol was added to control cells, which were grown in the absence of estrogen.

### Immunofluorescence

Cells were fixed in 4% paraformaldehyde in phosphate buffered saline (PBS) for 10 min at 4 °C. Fixed cells were exposed to a one-hour blocking step in 10% horse serum in 0.1% TritonX in PBS, before being stained with anti-SOX9 (ab5535; Millipore; 1:500) or anti-ERα (ab37438; Abcam; 1:500) and the nuclear marker DAPI (4′,6-diamidino-2-phenylindole in Fluoroshield mounting medium; ab104139; Abcam). SOX9 and ERα were visualized using Alexa-Fluor 555 anti-rabbit secondary antibody (A21428; Invitrogen; 1:500). Images were captured on a Nikon A1R confocal. At least five images from each individual culture within a treatment group were randomly chosen for analysis of SOX9. Using Image J software, we calculated Manders’ coefficient to determine the percentage of cytoplasmic SOX9. Mann-Whitney U tests were used to compare the percentage of cytoplasmic SOX9 between control and treated cells.

### RNA extraction, RT-PCR, and qPCR

RNA was extracted from NT2/D1 cells using the GenElute Mammalian Total RNA Miniprep Kit (Sigma Aldrich) as per the manufacturer’s instructions. Total RNA was DNase treated using Turbo DNA free (Ambion) as per the manufacturer’s instructions. Complementary DNA was produced using the Superscript III First Strand Synthesis System for reverse transcriptase (RT)-PCR (Invitrogen) according to the manufacturer’s instructions. Quantitative PCR was carried out on a Stratagene MX3000P system, using Quantitect SYBR Green PCR Master Mix (Qiagen) at 55 °C annealing temperature. mRNA levels of *SOX9, SRY, AMH, FGF9, PTGDS, FOXL2*, and *WNT4* were normalized against reference genes *CHMP2A* or *TBP* using the method described by Pfaffl [71]. Primer sequences used are provided in supplementary materials Table 1. Expression levels in control cell culture were designated as 1 and expression of each gene in the corresponding estrogen treated culture was expressed relative to control levels. Standard t-tests were used to determine any significant differences in gene expression between control and treated cells.

## Supplementary information

**Additional file 1.**

## Data Availability

All data generated or analysed during this study are included in this published article and its supplementary information files.

## Abbreviations

EDCs: Endocrine disrupting chemicals
DSDs: Differences of sexual development
NT2/D1: NTERA-2 clone D1
EE2: 17a-ethynylestradiol
SOX9: SRY-box transcription factor 9
AMH: Anti-mullerian hormone
FGF9: Fibroblast growth factor 9
PTGDS: Prostaglandin D synthase
WNT4: WNT family member 4
FOXL2: Forkhead box L2
ERα: Estrogen receptor α
