# Supplementary material for: Estrogen suppresses SOX9 and activates markers of female development in a human testis-derived cell line
Source: BMC Mol Cell Biol. 2020 Sep 15;21:66. doi: 10.1186/s12860-020-00307-9 (PMC7493336; doi:10.1186/s12860-020-00307-9)
Supplement: Supplementary file 1 — Additional file 1. [file 12860_2020_307_MOESM1_ESM.pdf]

Supplementary material

Table 1. Primer sequences used for qPCR

**Gene**	**Accession number**	**Forward primer (5’ – 3’)**	**Reverse primer (5’ – 3’)**
SOX9	NM_000346.3	ATCTGAAGAAGGAGAGCGAG	TCAGAAGTCTCCAGAGCTTG
SRY	NM_003140.2	TCAGCAAGCAGCTGGGATAC	AACTGCAATTCTTCGGCAGC
AMH	NM_000479.4	GCTGCCTTGCCCTCTCTAC	GAACCTCAGCGAGGGTGTT
FGF9	NM_002010.2	CTCCTGGGTTGACACCATCAT	CAGAATGCCAAATCGGCTG
PTGDS	NM_000954.5	CCTGCCCCAAACCGATAAGT	CAGAGACATCCAGAGCGTGG
FOXL2	NM_023067.3	GGACGGACCAATACGTGTGT	ACTGATAGCGGAGGAAACGC
WNT4	NM_030761.4	GTCTTCGCCGTCTTCTCAGC	GGAACTGGTACTGGCACTCC
CHMP2A	NM_014453.3	GGACCCTGTCGTCAACATCG	TGTGCCATCGAGTTGTTGGA
TBP	NM_003194.4	AGCGCAAGGGTTTCTGGTTT	CTGAATAGGCTGTGGGGTCA
